# Enhancing Outcomes in Knee and Hip Arthroplasty: A Multifaceted Approach

**DOI:** 10.3390/jcm14113651

**Published:** 2025-05-23

**Authors:** Nicholas David Clement

**Affiliations:** Edinburgh Orthopaedics, Royal Infirmary of Edinburgh, Edinburgh EH16 4SA, UK; nick.clement@nhs.scot

The prevalence of osteoarthritis continues to rise [1,2]. Arthroplasty is a cost-effective treatment for end-stage disease with excellent functional outcomes [3,4,5]. It is unsurprising that total hip arthroplasty was recognized as the most significant operation of the 20th century [6]. However, optimizing patient outcomes and their satisfaction after arthroplasty is complex [7,8,9]. Recent studies highlight the interplay between patient-specific factors [10], surgical techniques [11], prehabilitation strategies [12], and perioperative management in enhancing recovery [13] and long-term success [14,15].

One key area of advancement is prehabilitation and supported postoperative recovery [16,17]. Patane et al. [18] demonstrated the potential of tele-home prehabilitation, particularly neuromuscular electrostimulation, to enhance walking autonomy and quality of life in patients with hip and knee arthroplasty. Similarly, Terradas-Monllor et al. [19] found that preoperative multimodal physiotherapy reduces pain catastrophizing and improves post-surgical outcomes, emphasizing the importance of both psychological and physical preparation.

Weight management remains crucial in arthroplasty outcomes [20,21]. Maman et al. [22] reported that bariatric surgery before total knee arthroplasty (TKA) reduced perioperative complications, but raised concerns regarding higher revision rates. While weight reduction benefits overall health [23], further research is needed to refine strategies for obese patients undergoing TKA [24].

Objective functional assessments are gaining recognition for their predictive value [25]. Yücel et al. [26] validated the use of the 30-Second Sit-to-Stand and Timed Up-and-Go tests in forecasting function following TKA, reinforcing the role of multidisciplinary evaluations. Additionally, Matteo et al. [27] used machine learning to predict hospital length of stay in arthroplasty patients, illustrating the promise of artificial intelligence in surgical planning and resource optimization [28].

Surgical precision and alignment remain fundamental in arthroplasty success [29,30,31]. Studies by Diconi et al. [32] and Arai et al. [33] highlight the biomechanical importance of optimal implant positioning and the advantages of robotic-assisted surgery [34]. Yang et al. [35] demonstrated that functional alignment [36] outperformed modified kinematic alignment [37] in achieving knee balance and patient-reported outcomes when using robotic-assisted surgery, which is supported by data from a systematic review [38]. As technology advances, robotic assistance and refined alignment techniques may enhance implant longevity and functional restoration [34,38,39].

Postoperative pain management remains a challenge, with opioid use under scrutiny due to the risk of dependence and poor outcomes [40,41]. Niculae et al. [42] showed that transdermal fentanyl patches provided effective pain relief with fewer side effects. Gomez et al. [43] highlighted the superiority of nerve blocks over local infiltration anesthesia in reducing opioid dependence. The multimodal approach to analgesia continues to evolve, aiming for optimal pain control with minimal adverse effects [44].

Surgical decision-making between unicompartmental knee arthroplasty (UKA) and TKA is evolving [45,46]. While UKA potentially offers advantages such as shorter hospital stays and reduced costs, concerns over higher revision rates persist [47,48,49]. Bayram et al. [50] suggest that modern selection criteria for UKA patients, along with improved prosthetic designs, may enhance outcomes, necessitating further validation through high-performance patient-reported outcome measures such as the metabolic equivalent of task score [51] and the Forgotten Joint score [52].

Finally, patient perspectives must remain central to arthroplasty advancements [53,54,55]. Longo et al. [56] demonstrated the psychological burden of waiting for knee arthroplasty, emphasizing the need for comprehensive preoperative education and support. Addressing patient fears and expectations can significantly impact their satisfaction and recovery [57].

The field of hip and knee arthroplasty continues to evolve through technological innovations [58,59], refined surgical techniques [30], and patient-centered care models [60]. As research advances, a multifaceted approach integrating biomechanical precision [61], personalized risk assessment [62], enhanced rehabilitation [63], and optimal pain management [64] will be key to achieving superior outcomes for patients undergoing arthroplasty surgery.
